# Sample size and precision of estimates in studies of depression screening tool accuracy: A meta‐research review of studies published in 2018–2021

**DOI:** 10.1002/mpr.1910

**Published:** 2022-04-01

**Authors:** Elsa‐Lynn Nassar, Brooke Levis, Marieke A. Neyer, Danielle B. Rice, Linda Booij, Andrea Benedetti, Brett D. Thombs

**Affiliations:** ^1^ Lady Davis Institute for Medical Research Jewish General Hospital Montreal Quebec Canada; ^2^ Department of Psychiatry McGill University Montreal Quebec Canada; ^3^ Centre for Prognosis Research School of Medicine Keele University Staffordshire UK; ^4^ Department of Epidemiology Biostatistics, and Occupational Health McGill University Montreal Quebec Canada; ^5^ Department of Psychology McGill University Montreal Quebec Canada; ^6^ Department of Psychology Concordia University Montreal Quebec Canada; ^7^ CHU Sainte‐Justine Hospital Research Centre Montreal Quebec Canada; ^8^ Department of Medicine McGill University Montreal Quebec Canada; ^9^ Respiratory Epidemiology and Clinical Research Unit McGill University Health Centre Montreal Quebec Canada; ^10^ Department of Educational and Counselling Psychology McGill University Montreal Quebec Canada; ^11^ Biomedical Ethics Unit McGill University Montreal Quebec Canada

**Keywords:** bias, depression, diagnostic test accuracy, sample size, screening

## Abstract

**Objectives:**

Depression screening tool accuracy studies should be conducted with large enough sample sizes to generate precise accuracy estimates. We assessed the proportion of recently published depression screening tool diagnostic accuracy studies that reported sample size calculations; the proportion that provided confidence intervals (CIs); and precision, based on the width and lower bounds of 95% CIs for sensitivity and specificity. In addition, we assessed whether these results have improved since a previous review of studies published in 2013–2015.

**Methods:**

MEDLINE was searched from January 1, 2018, through May 21, 2021.

**Results:**

Twelve of 106 primary studies (11%) described a viable sample size calculation, which represented an improvement of 8% since the last review. Thirty‐six studies (34%) provided reasonably accurate CIs. Of 103 studies where 95% CIs were provided or could be calculated, seven (7%) had sensitivity CI widths of ≤10%, whereas 58 (56%) had widths of ≥21%. Eighty‐four studies (82%) had lower bounds of CIs <80% for sensitivity and 77 studies (75%) for specificity. These results were similar to those reported previously.

**Conclusion:**

Few studies reported sample size calculations, and the number of included individuals in most studies was too small to generate reasonably precise accuracy estimates.

## 1 INTRODUCTION

Major depression is a common and disabling disorder that accounts for more years of healthy life lost than any other medical condition (Lopez et al., 2006; Mathers et al., 2006; Moussavi et al., 2007; Whiteford et al., 2013). Depression screening has been proposed to identify individuals with unrecognized and untreated depression (Siu et al., 2016). Screening involves using depression symptom questionnaires to classify individuals as having positive or negative screens based on scoring above or below a cut‐off. Those above the cut‐off can be interviewed to determine if they have major depression, whereas those below the threshold are not further assessed. Whether screening should be implemented, however, is controversial (Thombs et al., 2017; Thombs et al., 2021). The United States Preventive Services Task Force has recommended screening for depression in general adult and perinatal populations (Siu et al., 2016). In contrast, the United Kingdom National Screening Committee (UK National Screening Committee, n.d.) and the Canadian Task Force on Preventive Health Care (Joffres et al., 2013) have recommended against depression screening due to a lack of direct evidence from trials that screening improves health outcomes and due to concerns about resource consumption and possible harms.

Studies of depression screening tool accuracy compare screening scores to depression status based on a reference standard diagnostic interview to determine an optimal cut‐off and to assess accuracy. This requires sample sizes with adequate numbers of participants with and without depression to generate precise estimates of sensitivity (the proportion of individuals with depression correctly identified by the screening tool) and specificity (the proportion of individuals without depression correctly ruled out by the screening tool). Accuracy studies with small samples sizes often fail to identify the most accurate cut‐off and overstate accuracy estimates for the cut‐offs they report (Bhandari et al., 2021). A simulation study based on real participant depression screening data from the Edinburgh Postnatal Depression Scale (EPDS) found that with samples of 100 participants, study‐specific optimal cut‐offs that maximized combined sensitivity and specificity ranged from ≥5 to ≥17 compared to the true population optimal cut‐off of ≥11 (Bhandari et al., 2021). On average, individual simulated studies overestimated sensitivity by 6.5 and underestimated specificity by 1.3% points. In contrast, with samples of 1000 participants, study‐specific optimal cut‐offs ranged from ≥8 to ≥13; on average, sensitivity and specificity were overestimated and underestimated by 1.4 and 1.0% points, respectively (Bhandari et al., 2021).

Many primary studies on depression screening tool accuracy are conducted with samples that are too small to accurately identify the best cut‐off to use and precisely estimate screening accuracy; few provide an a priori sample size calculation. A review of primary studies on the accuracy of depression screening tools published between 2013 and 2015 (*N* = 89) found that the median total sample size was 224, but the median number of depression cases was 37; only three of 89 studies (3%) reported an accurate sample size calculation, only 30 studies (34%) provided plausible confidence intervals (CIs) for accuracy estimates, and only seven studies (8%) had 95% CI widths for sensitivity ≤10% (Thombs & Rice, 2016). The included studies in that review, however, were published 6–8 years ago, and it is not known whether studies published more recently have improved.

The first objective of the present study was to review recently published studies of depression screening tool accuracy to assess the (1a) proportion that reported a correctly derived a priori sample size calculation, (1b) proportion that provided plausible CIs for accuracy estimates, (1c) precision of sensitivity and specificity estimates, and (1d) lower bounds of sensitivity and specificity CIs. We documented the lower bounds because they are often ignored in interpreting results from screening accuracy studies but represent plausible values that should be considered in making decisions about screening tools. The second objective was to assess whether these results have improved compared to the studies published from January 2013 to May 2015 that were included in the previous review (Thombs & Rice, 2016).

## 2 METHODS

This was a meta‐research review that evaluated primary research studies on depression screening tool accuracy published from January 2018 to May 2021 and compared results to those from the previous review (Thombs & Rice, 2016). Methods were based on those used in that review (Thombs & Rice, 2016). Prior to initiating the present study, a study protocol was developed and posted on the Open Science Framework (https://osf.io/5vmr4/).

### 2.1 Eligibility

Primary studies published in any language were eligible if they reported sensitivity and specificity estimates for one or more depression screening tools compared to depression classification based on a diagnostic interview. Primary studies were excluded if the reference standard was based on chart notes or a score above a threshold on another self‐report measure or rating scale. Primary studies that included only individuals in mental health treatment or seeking mental health services were also excluded since screening is conducted to identify individuals with unrecognized depression (Nassar et al., 2022; Rice & Thombs, 2016; Thombs et al., 2011, 2012, 2021).

### 2.2 Survey of recently published primary studies

We searched MEDLINE (PubMed interface) on May 21, 2021 for primary studies published January 1, 2018, or later, using the search terms (depress*[Title/Abstract] AND sensitivity [Title/Abstract] AND specificity [Title/Abstract]) AND (("2018/01/01" [Date ‐ Publication]: "3000" [Date ‐ Publication])), restricted to title or abstract. We included studies published in 2018 or later to be able to evaluate recent studies reflecting current research practices and published approximately 3–6 years since the 2015 review on this topic (Thombs & Rice, 2016). The PubMed search was conducted via the systematic review software DistillerSR (Evidence Partners, Ottawa, Canada), and citations were uploaded to the platform. To obtain the most recently published studies possible, we reviewed citations for eligibility backwards by PubMed identification number, starting with the most recent, and planning to stop when we obtained our targeted number of studies or when all citations in the study period were reviewed, whichever occurred first. Two investigators independently reviewed studies for eligibility. If either reviewer deemed a study potentially eligible based on title and abstract review, full‐text review was conducted, also independently by two reviewers. Any disagreements after full‐text review were resolved by consensus.

### 2.3 Sample size calculation

To determine the number of studies to target, we conducted a sample size calculation based on the precision of CIs of proportions calculated via the method of Agresti and Coull (1998). Based on the previous review of studies published from 2013 to 2015 (Thombs & Rice, 2016), we varied the proportion of interest from 3% (described a viable sample size calculation) to 34% (provided reasonably accurate CIs) and considered scenarios where the previously obtained proportions doubled, in case of improvements in current practices. We found that the maximum number of included studies needed to get CI widths smaller than 10% for providing a sample size calculation and <15% for reporting a plausible CI was 150 or fewer for all scenarios. Because the consequence of overpowering the study represented additional labor rather than risk to human participants, we aimed to include up to 160 studies, if possible, in the study period (S1 Appendix).

### 2.4 Data extraction

For all data extraction, one reviewer extracted the data from each included study, and a second reviewer verified the extracted data using the DistillerSR Quality Control function. Any discrepancies were resolved by consensus between the two reviewers and involving a third reviewer if necessary.

We assessed the proportion of studies that reported any sample size calculation and the proportion that reported a plausible precision‐based method to calculate sample size for estimating sensitivity and specificity. In addition, we assessed the proportion of studies that reported CIs around sensitivity and specificity estimates. If CIs were provided but were clearly incorrect and departed substantively from an appropriately calculated interval using standard methods, the study was coded as not providing plausible CIs.

We extracted information using a standardized data extraction form via DistillerSR. For each primary study, we extracted the (1) first author's last name; (2) publication year; (3) journal and its most recent impact factor prior to or including the publication year; (4) country; (5) screening tool(s) evaluated; (6) reference standard; (7) study population; (8) number of participants; (9) number of depression cases; (10) reporting of an appropriately derived a priori sample size calculation; (11) cut‐off for data extraction; (12) sensitivity and specificity estimates with 95% CIs, if provided; and (13) whether the study reported compliance with the STAndards for Reporting Diagnostic accuracy studies statement (STARD; Bossuyt et al., 2015).

For primary studies with multiple screening tools or reference standards, we only extracted data for the first screening tool and reference standard combination listed in the abstract or article text, prioritizing the abstract. When results were reported for multiple cut‐off thresholds, we extracted data for the cut‐off prioritized by the authors as the “primary”, “standard”, or “optimal” cut‐off or, if not specified, for the first cut‐off for which results were reported in the abstract or article text, prioritizing the abstract.

## 3 ANALYSIS

We first (objective 1a) estimated the proportion of studies that reported an a priori sample size calculation, including the proportion that described an appropriate precision‐based method to calculate sample size for sensitivity and specificity estimates. Second (objective 1b), we estimated the proportion of studies that provided plausible CI estimates for sensitivity and specificity. Third (objective 1c), we classified 95% CI widths for sensitivity and specificity as between 0% and 5%, 6%–10%, 11%–20%, 21%–30%, 31%–40%, 41%–50%, or >50%, and we estimated the proportion in each category. Fourth (objective 1d), for sensitivity and specificity, we estimated the proportion of studies with lower 95% CI bounds <80%, 80%–84%, 85%–89%, 90%–94%, and ≥95%. If 95% CIs were not provided, we estimated CIs based on data provided in the publication, using an approximation method for interval estimation of binomial proportions recommended by Agresti and Coull (1998). If 95% CIs were provided but were clearly erroneous due to substantial deviation from plausible values, we also estimated the 95% CI. Finally, we estimated the proportion of studies that reported compliance with the STARD statement, which recommends conducting a priori sample size calculations (Bossuyt et al., 2015).

In addition, we conducted sensitivity analyses that included only journals with impact factor ≥3 for the year of publication, as was done in the previous review (Thombs & Rice, 2016). This allowed us to explore whether studies published in journals with higher impact factors were more likely to report an appropriately derived a priori sample size calculation; to report CIs, and, if so, had narrower intervals than studies published in journals with a lower impact factor.

To assess whether these results have improved since 2015 (objective 2), we compared the proportions found in the present study to the proportions reported by Thombs and Rice (2016) using a test for differences in proportions and a 95% CI around the difference.

## 4 RESULTS

### 4.1 Search results

**FIGURE 1:**
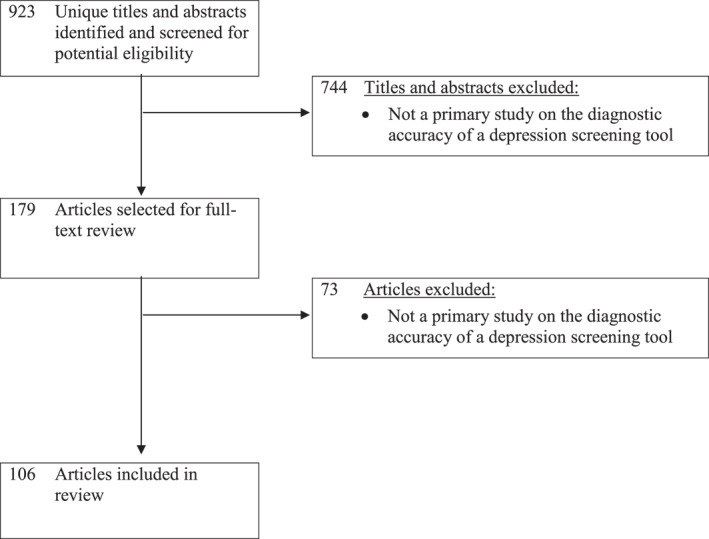
Flow diagram of selection of primary studies that evaluated the diagnostic accuracy of depression screening tools

The database search yielded 923 unique titles and abstracts. Of these, 744 were excluded after title and abstract review and 73 after full‐text review, leaving 106 eligible primary studies (Figure 1). The 106 studies included sample sizes between 38 and 6700 (median = 243); the number of depression cases ranged from six to 454 (median = 37). Most studies were from Asia (*N* = 32; 30%), Europe (*N* = 28; 26%), North America (*N* = 17; 16%), or Africa (*N* = 14; 13%). The most common depression screening tools were the Patient Health Questionnaire (Kroenke et al., 2001; Spitzer et al., 1999; any version, 35 studies), Geriatric Depression Scale (Yesavage et al., 1982; any version; nine studies), EPDS (Cox et al., 1987; any version; eight studies), and Center for Epidemiologic Studies Depression Scale (Radloff, 1977; any version; seven studies). There were 59 studies (56%) from journals with impact factor ≥3. Included study characteristics are shown in S2 Appendix.

### 4.2 Sample size calculations

Twenty‐seven of 106 primary studies (25%; 95% CI, 18%–35%) mentioned a sample size calculation. However, only 12 (11%; 95% CI, 7%–19%) described a plausible precision‐based method, including eight of 59 in journals with impact factor ≥3 (14%; 95% CI, 7%–25%). Reasons for why the remaining 15 of 27 studies that reported a sample size calculation were classified as not reporting a plausible precision‐based method are provided in S3 Appendix.

### 4.3 Reporting of confidence intervals

Of the 106 primary studies, 36 (34%; 95% CI, 26%–43%) reported 95% CIs, but one study reported implausible intervals (e.g., CI endpoint >100%). Thus, 35 studies (33%; 95% CI, 25%–42%) published reasonably accurate 95% CIs, including 22 of 59 in journals with impact factor ≥3 (37%; 95% CI, 26%–50%). See S4 Appendix.

### 4.4 Precision of confidence intervals

As shown in Table 1, among the 103 studies for which 95% CIs were provided or could be estimated, only seven (7%; 95% CI, 3%–13%) had widths ≤10% for sensitivity, whereas 58 (56%; 95% CI, 47%–66%) had widths ≥21%, and 27 (26%; 95% CI, 19%–36%) had widths ≥31%. Among the 59 studies from journals with impact factor ≥3, four (7%; 95% CI, 3%–16%) had widths ≤10%, 33 (56%; 95% CI, 43%–68%) had widths ≥21%, and 17 (29%; 95% CI, 19%–41%) had widths ≥31%.

**TABLE 1:** Precision of sensitivity and specificity among 103 primary studies for which 95% confidence intervals (CIs) were published or could be calculated

	All studies		Studies published in journals with impact factor ≥3	
Width of 95% confidence interval	Sensitivity *N* (%) studies	Specificity *N* (%) studies	Sensitivity *N* (%) studies	Specificity *N* (%) studies
0%–5%	2 (2)	13 (13)	2 (3)	9 (15)
6%–10%	6 (6)	31 (30)	3 (5)	14 (24)
11%–20%	37 (36)	50 (49)	19 (32)	31 (53)
21%–30%	31 (30)	9 (9)	16 (27)	3 (5)
31%–40%	17 (17)	0 (0)	12 (20)	0 (0)
41%–50%	8 (8)	0 (0)	4 (7)	0 (0)
>50%	2 (2)	0 (0)	1 (2)	0 (0)
Total	103 (100)	103 (100)	59 (100)	59 (100)

For specificity, there were 38 studies (37%; 95% CI, 28%–47%) with 95% CI widths ≤10%; nine (9%; 95% CI, 5%–16%) had widths 21% or greater. Among the 59 studies from journals with impact factor ≥3, 21 (36%; 95% CI, 25%–48%) had widths ≤10%, and three (5%; 95% CI, 2%–14%) had width ≥21% (see Table 1).

### 4.5 Lower bounds of confidence intervals

As shown in Table 2, the lower bound of 95% CIs was <80% for 84 (82%; 95% CI, 73%–88%) studies for sensitivity and 77 (75%; 95% CI, 66%–82%) studies for specificity. Only two studies (2%; 95% CI, 0.5%–6%) had a lower bound ≥90% for sensitivity and only four (4%; 95% CI, 2%–10%) for specificity. Results were similar for studies published in journals with impact factor ≥3 (see Table 2).

**TABLE 2:** Lower bounds of 95% confidence intervals (CIs) among 103 primary studies for which 95% CIs were published or could be calculated

	All studies		Studies published in journals with impact factor ≥3	
Lower bound of 95% confidence interval	Sensitivity *N* (%) studies	Specificity *N* (%) studies	Sensitivity *N* (%) studies	Specificity *N* (%) studies
<80%	84 (82)	77 (75)	48 (81)	43 (73)
80%–84%	14 (14)	13 (13)	6 (10)	6 (10)
85%–89%	3 (3)	9 (9)	2 (3)	5 (8)
90%–94%	2 (2)	4 (4)	1 (2)	3 (5)
≥95%	0 (0)	0 (0)	0 (0)	0 (0)
Total	103 (100)	103 (100)	59 (100)	59 (100)

### 4.6 Compliance with the STARD statement

Among the 106 included studies, four (4%; 95% CI, 1%–9%) reported compliance with the STARD statement; all four studies were published in journals with impact factors ≥3.

### 4.7 Comparison to studies published 2013–2015

The proportion of studies that reported an appropriate precision‐based method to calculate sample size for sensitivity and specificity estimates improved from 3% in studies published in 2013%‐2015% to 11% in studies in the review of studies published in 2018–2021, an improvement of 8% (95% CI, 1%–15%; Thombs & Rice, 2016).

The proportion of studies that reported reasonably accurate CIs around accuracy estimates was 34% in both 2013–2015 and more recent studies (0%, 95% CI, 0%–14%). For sensitivity, the proportion of studies that reported CI widths ≤10% decreased from 8% to 7% (1%; 95% CI, 0%–6%). The proportion that reported CI widths ≥21% decreased from 62% to 56%, (6%; 95% CI, 0%–19%); the proportion that reported CI widths ≥31% increased from 23% to 26% (3%; 95% CI, 0%–15%). For specificity, the proportion of studies that reported CI widths ≤10% decreased from 45% to 37% (8%; 95% CI, 0%–6%); the proportion that reported CI widths ≥21% increased from 7% to 9% (2%; 95% CI, 0%–9%; Thombs & Rice, 2016).

For sensitivity, the proportion of studies with a lower bound of 95% CIs <80% was 84% in studies published in 2013%–2015% and 82% in more recently published studies (2%; 95% CI, 0%–9%); the proportion with a lower bound ≥90% increased from 1% to 2% (1%; 95% CI, 0%–5%). For specificity, the proportion of studies with a lower bound of 95% CIs <80% increased from 66% to 75% (9%; 95% CI, 0%–22%); the proportion with a lower bound ≥90% decreased from 6% to 4% (2%; 95% CI, 0%–4%; Thombs & Rice, 2016).

## 5 DISCUSSION

Among the 106 recently published studies on the diagnostic accuracy of depression screening tools that we surveyed, only 12 (11%) described a viable method for a precision‐based sample size calculation. Only 35 studies (33%) provided accurate CIs for estimates of sensitivity and specificity. Precision was generally poor, particularly for sensitivity. For sensitivity, only 7% of studies had 95% CIs with widths of 10% or less, whereas 57% had intervals with widths of more than 20%. For specificity, 37% of studies had 95% CIs with widths of 10% or less, and only 9% had widths of more than 20%. Lower bounds of 95% CIs were less than 80% for 82% of studies for sensitivity and 75% of studies for specificity. Results were similar when only the 59 (56%) studies published in journals with impact factors of at least three were evaluated. The proportion of studies that described a viable method for a precision‐based a priori sample size calculation improved from 3% in studies published in 2013–2015 (Thombs & Rice, 2016) to 11% in recently published studies, but this is still very low. The precision and lower bounds of CIs around sensitivity and specificity estimates did not change between studies published in 2013–2015 (Thombs & Rice, 2016) and recently published studies.

To ensure that studies generate reasonably precise estimates of sensitivity and specificity, investigators should consider the precision that is needed for use in clinical practice and should calculate the sample size required to achieve that level of precision. However, we found that only 11% of studies described a viable method for a precision‐based sample size (an additional 15% reported incorrect sample size calculations). The STARD (Bossuyt et al., 2015) statement includes an a priori sample size calculation as an essential reporting item. Among the studies we reviewed, indicated compliance with STARD was found to be very low; only 4% of studies cited the STARD guideline. This points to the need for researchers to utilize STARD guidance in designing and reporting their studies and for peer reviewers and editors to insist on compliance.

In cases where study authors cannot recruit sufficient participants to generate precise estimates of accuracy, it may still be possible for such studies to collectively provide important information. Decisions about screening tools and cut‐offs to use in practice should be derived using large, high‐quality meta‐analyses. Thus, if primary studies include small samples but report all results across all cut‐offs, even if in appendices, as opposed to selectively reporting only cut‐offs that performed well in their study (Levis et al., 2017; Neupane et al., 2021), they can contribute meaningfully to the overall evidence base. Ideally, study authors should also make their individual participant data available for pooling with other studies, including key variables such as participant characteristics, so that subgroup analyses may be performed. Meta‐analyses of individual participant data, which combine datasets from primary studies, can assess all cut‐offs for all participants and, therefore, provide a solution to selective reporting (Levis et al., 2019). Large individual meta‐analyses of individual participant data have been conducted with some of the most commonly used depression screening tools, such as the EPDS (Levis, Negeri et al., 2020), the Hospital Anxiety and Depression Scale – Depression subscale (Wu et al., 2021), and the Patient Health Questionnaire‐2 and ‐9 (Levis et al., 2019; Levis, Sun et al., 2020; Negeri et al., 2021).

If authors do conduct studies with small sample sizes and low precision, they should underline the importance of accruing data from well‐conducted studies, but they should avoid drawing strong conclusions about the optimal cut‐off to use or how accurate the tool is in their study population. Indeed, a simulation study with the EPDS (Bhandari et al., 2021) reported that only about a third of studies with total samples of 100‐200 participants identified the correct population optimal cut‐off and that this increased to just over 50% with 500 participants. Thus, drawing conclusions for practice with small sample sizes may mislead users about the accuracy of depression screening tools, in general, or for use in specific populations.

The results of the present study should be interpreted with respect to the following limitations. First, we were able to identify 106 eligible studies in the review period, which was fewer than our targeted sample size of 160, which we set to obtain 95% CI widths <15%. Nonetheless, all 95% CI widths were <20%. Second, we only searched the MEDLINE database for eligible studies, which could have led us to not identify some eligible studies. However, searching only MEDLINE for studies of diagnostic test accuracy, generally, has been shown to not influence summary estimates in meta‐analyses (Van Enst et al., 2014). Restricting searches to MEDLINE has been shown to capture almost all (approximately 95%) of the eligible studies included in meta‐analyses on the accuracy of depression screening tools (Rice, Kloda, Levis, Kingsland, & Thombs, 2016). Thus, it is unlikely that our main findings would have changed if other databases had been searched. Third, the included studies were published in a wide range of journals and were conducted on a wide range of depression screening tools. However, our results remained unchanged when only studies from journals with an impact factor ≥3 were evaluated.

In summary, we found that 11% of primary studies on the diagnostic accuracy of depression screening tools published since 2018 appropriately reported a viable precision‐based method for calculating an a priori sample size, an 8% improvement since the last review of studies published in 2013–2015 (Thombs & Rice, 2016). The proportion of studies that provided CIs to quantify the precision of accuracy estimates remained unchanged since the last review (Thombs & Rice, 2016), at just over a third of studies. Overall, sample sizes of most included studies were too small to generate precise estimates of accuracy; over half of studies had 95% CIs for sensitivity that were wider than 20%. Future studies on the diagnostic accuracy of depression screening tools should conduct precision‐based a priori sample size calculations to either attain desired precision levels or to understand limitations prior to initiating a study. Reports of study results should comply with the STARD guideline, and conclusions should fully consider the imprecision of estimates of screening accuracy.

## CONFLICT OF INTERESTS

All authors completed the International Committee of Medical Journal Editors uniform disclosure form and declared no support from any organization for the submitted work; no financial relationships with any organizations that might have an interest in the submitted work in the previous 3 years. All authors declare no other relationships or activities that could appear to have influenced the submitted work.

## AUTHOR CONTRIBUTIONS

Elsa‐Lynn Nassar, Brooke Levis, Danielle B. Rice, Linda Booij, Andrea Benedetti, and Brett D. Thombs contributed to the conception and design of the study. Elsa‐Lynn Nassar, Brooke Levis, Marieke A. Neyer, and Brett D. Thombs contributed to data extraction, coding, and evaluation of included studies. Elsa‐Lynn Nassar, Brooke Levis, Andrea Benedetti, and Brett D. Thombs contributed to the data analysis plan, and Elsa‐Lynn Nassar conducted the analyses. Elsa‐Lynn Nassar drafted the manuscript, and Brooke Levis, Marieke A. Neyer, Danielle B. Rice, Linda Booij, Andrea Benedetti, and Brett D. Thombs provided critical reviews and approved submission of the final manuscript.

## Supporting information

Supplementary Material 1

## Data Availability

All data from the study are available in table format in the supplementary materials.
