# Supplementary material for: Sample size and precision of estimates in studies of depression screening tool accuracy: A meta‐research review of studies published in 2018–2021
Source: Int J Methods Psychiatr Res. 2022 Apr 1;31(2):e1910. doi: 10.1002/mpr.1910 (PMC9159687; doi:10.1002/mpr.1910)
Supplement: Supplementary file 1 — Supplementary Material 1 [file MPR-31-e1910-s001.docx]

**S1 Appendix: Sample size calculation**

**Scenarios based on proportions reported by Thombs and Rice (2016)**

|  |  |  |  | **Study counts** | |  |  |  |  |
| --- | --- | --- | --- | --- | --- | --- | --- | --- | --- |
| **Scenario** | **Proportion of interest** | **Total *N*** | **Percent “Yes”** | ***N* = Yes** | ***N* = No** | **Proportion** | **95% low** | **95% high** | **Width** |
| **Specified *N* calculation** (proportion found: 3%) | 0.03 | 63 | 3 | 2 | 61 | 3.17% | 0.87% | 10.86% | 9.98% |
| **Reported plausible CI** (proportion found: 34%) | 0.34 | 150 | 34 | 51 | 99 | 34.00% | 26.90% | 41.90% | 14.99% |
|  | 0.34 | 342 | 34 | 116 | 226 | 33.92% | 29.10% | 39.09% | 9.99% |

**Scenarios where the previously obtained proportions were doubled**

|  |  |  |  | **Study counts** | |  |  |  |  |
| --- | --- | --- | --- | --- | --- | --- | --- | --- | --- |
| **Scenario** | **Proportion of interest** | **Total *N*** | **Percent “Yes”** | ***N* = Yes** | ***N* = No** | **Proportion** | **95% low** | **95% high** | **Width** |
| **Specified *N* Calculation** (proportion estimated: 6%) | 0.06 | 90 | 6 | 5 | 85 | 5.56% | 2.40% | 12.35% | 9.96% |
| **Reported plausible CI** (proportion estimated: 68%) | 0.68 | 145 | 68 | 99 | 46 | 68.28% | 60.31% | 75.30% | 14.98% |
|  | 0.68 | 332 | 68 | 226 | 106 | 68.07% | 62.88% | 72.86% | 9.98% |

**S2 Appendix: Characteristics of primary studies of the diagnostic accuracy of depression screening tools**

| **First author,  last name** | **Year** | **Journal** | **2020 impact factor** | **Country(ies)** | **Population** | ***N* screened and  interviewed** | ***N* cases** | **Screening tool and cutoff threshold used for precision estimates** |
| --- | --- | --- | --- | --- | --- | --- | --- | --- |
| Akena | 2018 | Br J Psychiatry | 9.3 | Uganda; South Africa | Patients in low literacy settings | 343 | 78 | AViDI-18 (16) |
| Alves | 2019 | Midwifery | 2.4 | Portugal | Perinatal women | 140 | 6 | PDPI-R (4.5) |
| Arturo Cassiani-Miranda | 2021 | Gen Hosp Psychiatry | 3.2 | Colombia | Adult primary care patients | 243 | 51 | HADS-D (4) |
| Aslan | 2020 | Front Psychiatry | 4.2 | Chile | Self-dependent primary care outpatients aged 65 to 80 | 577 | 21 | PHQ-9 (6) |
| Baldellou Lopez | 2021 | J Psychosom Res | 3 | United Kingdom | Adults with dissociative seizures in the previous 8 weeks | 368 | 114 | PHQ-9 (13) |
| Ballester | 2019 | PLoS One | 3.2 | Spain | First year university students aged 18 to 24 | 287 | 26 | WMH-ICS online screening scale (15) |
| Baumgartner | 2019 | Psychiatr Prax | 1.5 | Austria | Individuals aged 18 to 65 from the general population | 504 | 39 | GDS-15 (2/3) |
| Bautovich | 2018 | Australas Psychiatry | 1.4 | Australia | Patients aged over 18 years with chronic haemodialysis | 45 | 6 | BDI (18) |
| Bernstein | 2018 | Inflamm Bowel Dis | 5.3 | United States | Individuals with inflammatory bowel disease aged 18 years or older | 242 | 21 | PHQ-2 (3) |
| Bhana | 2019 | S Afr Med J | 1.6 | South Africa | Outpatients aged 18 years or older attending primary healthcare clinics | 1206 | 57 | PHQ-2 (3) |
| Binagwaho | 2021 | BMC Pediatr | 2.1 | Rwanda | Children with HIV aged 7 to 14 | 296 | 42 | CDST (6) |
| Blanco | 2019 | Aging Ment Health | 3.7 | Spain | Non-professional caregivers | 294 | 30 | CD-RISC 10 (23) |
| Borghero | 2018 | Rev Med Chil | 0.6 | Chile | Adolescents aged 15 to 19 years | 245 | 210 | PHQ-9 (11) |
| Butnoriene | 2018 | BMC Psychiatry | 3.6 | Lithuania | Individuals aged 45 years or older with and without metabolic syndrome | 1115 | 201 | HADS-D (8) |
| Cassiani-Miranda | 2021 | Rev Colomb Psiquiatr | NA | Colombia | Primary care patients aged 18 to 65 years | 243 | 53 | PHQ-9 (7) |
| Chenneville | 2019 | J Affect Disord | 4.8 | United States | People with HIV aged 12 to 25 years receiving care in an integrated care setting | 121 | 37 | PHQ-9 (5.5) |
| Clover | 2018 | Qual Life Res | 4.1 | Australia | Outpatients with cancer | 132 | 18 | PROMIS-D-CAT (53) |
| Colomo | 2021 | Arch Bronconeumol | NA | Spain | Adult patients with bronchiectasis | 52 | 18 | HADS-D (9) |
| Cruzado | 2018 | Support Care Cancer | 3.6 | Spain | Adult cancer patients | 130 | 6 | HADS-T (10) |
| Cumbe | 2020 | BMC Psychiatry | 3.6 | Mozambique | Prenatal, postnatal, and general outpatients | 502 | 43 | PHQ-9 (9) |
| Dajpratham | 2020 | BMC Psychiatry | 3.6 | Thailand | First-time stroke patients aged 45 and older | 115 | 23 | PHQ-9 (6) |
| Degefa | 2020 | BMC Psychiatry | 3.6 | Ethiopia | Adult outpatients with cancer | 163 | 25 | PHQ-9 (4) |
| Durmaz | 2018 | North Clin Istanb | NA | Turkey | Older adults (aged 65 or over) outpatients visiting a geriatric clinic | 329 | 81 | GDS-15 (5) |
| Eriksen | 2019 | J Affect Disord | 4.8 | Norway | Home-dwelling adults aged 60 years or older | 194 | 56 | GDS-5 (2) |
| Figueiredo-Duarte | 2019 | Aging Ment Health | 3.7 | Portugal | Institutionalized adults aged 60 or older | 186 | 20 | GDS (5/6) |
| Fuseekul | 2021 | Child Adolesc Psychiatry Ment Health | 3 | Thailand | Secondary school students aged 12-18 | 103 | 31 | MFQ (28) |
| Gallis | 2018 | PeerJ | 3 | Pakistan | Community-based pregnant women | 1731 | 454 | PHQ-9 (10) |
| Getting it Right  Collaborative Group | 2019 | Med J Aust | 7.7 | Australia | Adults (18 years or older) identifying as Indigenous Australians | 500 | 108 | PHQ-9 (10) |
| Ghazisaeedi | 2021 | Int J Ment Health Addict | 3.8 | Iran | Undergraduate medical students | 400 | NR | PHQ-9 (5) |
| Gholizadeh | 2019 | Contemp Nurse | 1.8 | Iran | Coronary artery disease inpatients aged 20 or above | 150 | 33 | PHQ-9 (7) |
| Gouweloos-Trines | 2019 | Eur J Psychotraumatol | 4.1 | Netherlands | Survivors of an airplane crash aged 14 years or older | 38 | 6 | PHQ-2 (3) |
| Green | 2018 | J Affect Disord | 4.8 | Kenya | Perinatal women (pregnant and postpartum) | 192 | 10 | EPDS (16) |
| Guerin | 2018 | J Affect Disord | 4.8 | United States | Adults aged 18 to 54 versus 55 to 80 | 311 | 117 | GDS (5) |
| Hamers | 2018 | J Appl Res Intellect Disabil | 2.7 | Netherlands | People with intellectual disabilities aged 18 to 49 years | 43 | 8 | ADAMS -  Depressive Mood Subscale (14) |
| Herizchi | 2020 | Health Promot Perspect | 0.6 | Iran | Adults 60 years and older | 387 | 44 | GDS (6) |
| Hirt | 2020 | J Affect Disord | 4.8 | Netherlands; Germany | Hospitalized patients with a diagnosis of ischemic stroke or intracerebral hemorrhage | 93 | 17 | DePreS (0) |
| Hitchon | 2020 | Arthritis Care Res | 4.8 | Canada | Outpatients with rheumatoid arthritis aged 18 or older | 150 | 17 | PHQ-2 (3) |
| Housen | 2018 | Transcult Psychiatry | 2.2 | India | Adult general medical outpatients aged 18 years or older | 290 | 81 | HSCL-Depression Scale (1.57) |
| Indu | 2018 | Asian J Psychiatr | 3.5 | India | Women aged 18-60 years in primary care | 228 | 57 | PHQ-9 (9) |
| Jahn | 2018 | Psychiatr Prax | 1.5 | Austria | Adults from the general population | 508 | 37 | CES-D (9/10) |
| Jokelainen | 2019 | Scand J Prim Health Care | 2.6 | Finland | Older adults born in 1935 | 505 | 24 | SDS (39) |
| Kagee | 2020 | Gen Hosp Psychiatry | 3.2 | South Africa | Patients receiving outpatient HIV care | 688 | 170 | CESD-R (27) |
| Karam | 2018 | Alzheimers Dement  (Amst) | 21.6 | Lebanon | Individuals aged between 63 and 101 years | 56 | 10 | A-CSDD (13) |
| Kim | 2020 | Seizure | 3.2 | Korea | Outpatients aged 18 or older with epilepsy | 213 | 48 | PHQ-9 (9.5) |
| Kokoszka | 2020 | Prim Care Diabetes | 2.5 | Poland | Outpatients and inpatients with Type 2 diabetes aged 18 or older | 101 | 35 | BDI (13) |
| Kwan | 2019 | Semin Arthritis Rheum | 5.5 | Canada | Adult outpatients with systemic lupus erythematosus | 159 | 23 | CES-D (16) |
| Kyranou | 2020 | BMC Psychiatry | 3.6 | Greece | Outpatients receiving chemotherapy for solid tumours | 152 | NR | DT (4) |
| Lafont | 2021 | Oncologist | 5 | France | Inpatients and outpatients aged 70 or older with solid or hematological cancers | 830 | 208 | GDS-4 (1) |
| Legha | 2020 | Confl Health | 2.7 | Haiti | Transitional age youth (18-22) attending schools | 120 | 9 | ZLDSI (12) |
| Liu | 2019 | Neuropsychiatr Dis Treat | 2.6 | Taiwan | Outpatients with chronic low back pain | 225 | 21 | DSSS-DS (15) |
| Loades | 2020 | Eur Child Adolesc Psychiatry | 4.8 | England | Adolescents (12-18 years) with Chronic Fatigue Syndrome/Myalgic Encephalomyelitis | 164 | 33 | RCADS-D (14) |
| Lydsdottir | 2019 | Midwifery | 2.4 | Iceland | Pregnant women | 474 | NR | EPDS (11) |
| Macêdo | 2018 | Lupus | 2.9 | Brazil | Patients with systemic lupus erythematosus | 108 | 21 | BDI (13) |
| Marrie | 2018 | Mult Scler Relat Disord | 4.3 | Canada | Outpatients with multiple sclerosis aged 18 years or older, attending a multiple sclerosis clinic | 253 | 26 | PHQ-9 (10) |
| Martínez | 2020 | J Clin Psychol | 2.9 | Chile | Mothers 18 years and older with infants aged 2-6 months | 298 | 63 | EPDS-3 (8/9) |
| Massai | 2018 | Parkinsons Dis | 2.7 | Italy | Patients with Parkinson's disease | 74 | 23 | GDS (15-16) |
| Matthey | 2019 | J Affect Disord | 4.8 | Australia | Pregnant women attending antenatal clinic | 247 | 7 | MGMQ (Lower Bother Impact threshold) |
| McCartney | 2020 | Epilepsy Behav | 2.9 | Australia | Adult inpatients aged 18 to 77 years with epilepsy or psychogenic non-epileptic seizure | 485 | 115 | HADS-D (7) |
| Mohsin | 2021 | Int J Environ Res Public Health | 3.4 | Pakistan | Pregnant women and mothers with children aged 3 years or less | 425 | 100 | CIDT-MD (3.5) |
| Molebatsi | 2020 | BMC Psychiatry | 3.6 | Botswana | Adult primary care patients | 257 | 105 | PHQ-9 (9) |
| Muramatsu | 2018 | Gen Hosp Psychiatry | 3.2 | Japan | Primary care patients | 284 | 93 | PHQ-9 (10) |
| Nabbe | 2019 | PLoS One | 3.2 | France | General practice patients aged 18 or older | 142 | 73 | HSCL-25 (1.75) |
| Navarrete | 2019 | Salud Publica Mex | 2 | Mexico | Women during  pregnancy | 210 | NR | Whooley questions (NR) |
| Park | 2020 | Front Psychol | 3 | Korea | Adults recruited online and during visit to hospital (patients, visitors, staff) | 1145 | 96 | BDI-II (23) |
| Peng | 2020 | Dermatol Ther | 2.9 | China | Outpatients with acne aged 18 to 24 years | 258 | 47 | PHQ-9 (9) |
| Rancans | 2018 | Ann Gen Psychiatry | 3.5 | Latvia | Primary care outpatients aged 18 years or older | 1467 | 150 | PHQ-9 (8) |
| Rashid | 2021 | Epilepsy Behav | 2.9 | India | Adult outpatients with epilepsy | 449 | 180 | NDDI-E (12) |
| Rashid | 2019 | Epilepsy Behav | 2.9 | India | Outpatients with epilepsy aged 18 or older | 217 | 90 | NDDI-E (12) |
| Recklitis | 2020 | Cancer | 6.9 | United States | Adult cancer survivors aged 18 to 40 years | 249 | 32 | PROMIS-D-SF (53.2) |
| Risal | 2019 | J Nepal Health Res Counc | NA | Nepal | Adult outpatients aged 60 years or older | 106 | 51 | GDS-15 (5/6) |
| Rodríguez-Mayoral | 2018 | Palliat Support Care | 2.3 | Mexico | Outpatients aged 18 and older with advanced cancer receiving palliative care | 70 | 14 | BEDS (5) |
| Saal | 2018 | AIDS Care | 2.3 | South Africa | Individuals aged 18 years or older seeking HIV testing | 500 | 72 | BDI-I (20) |
| Saldivia | 2019 | Rev Med Chil | 0.6 | Chile | Primary care patients aged 18 to 75 years | 1738 | 187 | PHQ-9 (7) |
| Sampasa-Kanyinga | 2018 | PLoS One | 3.2 | Canada | Military personnel | 6700 | 208 | K10 (10) |
| Sasaki | 2019 | J Obstet Gynaecol Res | 1.7 | Japan | Women 4 days to 1 month postpartum | 80 | 9 | EPDS (10) |
| Scoppetta | 2021 | J Affect Disord | 4.8 | Colombia | Adult primary care patients | 243 | 52 | PHQ-2 (2) |
| Searle | 2019 | Assessment | 4.7 | Regular-serving military personnel | Australia | 1730 | 74 | K10 (19) |
| Shaheen | 2019 | Am J Mens Health | 2.8 | Saudi Arabia | Fathers of newborns aged up to 6 months | 57 | 9 | EPDS (8/9) |
| Shih | 2020 | Seizure | 3.2 | Taiwan | Inpatients and outpatients with epilepsy aged 20 or older | 109 | 20 | NDDI-E (15) |
| Silagadze | 2019 | Epilepsy Behav | 2.9 | Georgia | Adult outpatients with epilepsy | 130 | 31 | NDDI-E (16) |
| Smith Fawzi | 2019 | Neurol Psychiatry Brain Res | NA | Tanzania | Primary care patients aged 18 years and older | 174 | 18 | PHQ-9 (9) |
| Smith-Nielsen | 2018 | BMC Psychiatry | 3.6 | Denmark | Women aged 18 or older who were 2 to 10 months postpartum | 324 | 118 | EPDS (11) |
| Tan | 2018 | J Am Psychiatr Nurses Assoc | 2.4 | Singapore | Adult outpatients aged 65 years or older attending a general geriatric outpatient clinic | 77 | 6 | Emoticon scale (5) |
| Tran | 2019 | J Affect Disord | 4.8 | Indonesia | Adolescents (aged between 16 and 18 years) attending senior schools | 189 | NR | CESD-R (20) |
| Tschorn | 2019 | Psychiatr Prax | 1.5 | Germany | Patients with coronary heart disease | 1019 | 83 | HADS-D (7) |
| Udedi | 2019 | BCM Psychiatry | 3.6 | Malawi | Outpatients with type-2 diabetes | 323 | 58 | PHQ-9 (9) |
| Urtasun | 2019 | BMC Psychiatry | 3.6 | Argentina | Adults attending primary care or mental health clinics | 169 | 102 | PHQ-9 (8) |
| van Heyningen | 2019 | Glob Ment Health | 3.5 | South Africa | Pregnant women aged 18 years or older receiving outpatient antenatal care | 376 | 81 | Whooley + EPDS + PHQ-9 (1) |
| van Heyningen | 2018 | PLos One | 3.2 | South Africa | Pregnant women attending a primary care antenatal clinic | 376 | 81 | EPDS (14) |
| Vázquez | 2019 | J Affect Disord | 4.8 | Spain | Pregnant women | 569 | 40 | EPDS (9) |
| Vogeli | 2018 | Res Nurs Health | 2.2 | United States | Mothers of 4- to 15-month-old infants | 238 | 36 | PDSS (80) |
| Vrublevska | 2018 | Nord J Psychiatry | 2.2 | Latvia | Adult (aged 18 years or older) primary care patients | 272 | 37 | PHQ-9 (10) |
| Wang | 2019 | Ther Apher Dial | 1.8 | China | Patients aged 18 years or older undergoing maintenance hemodialysis | 112 | 24 | DI-MHD (25) |
| Wang | 2018 | J Geriatr Psychiatry Neurol | 2.7 | United States | Older adult outpatients with ischemic heart disease and prior stroke | 147 | 35 | CES-D (10) |
| Weihs | 2018 | Psychooncology | 3.9 | United States | Patients with breast cancer | 82 | 14 | DRQ-7 (6) |
| Williams | 2021 | Assessment | 4.7 | United States | Adults with autism spectrum disorder aged 18 to 45 years | 66 | 24 | BDI-II (14) |
| Williams | 2020 | Neurol Clin Pract | NA | Canada | Outpatients with neurologic disorders aged 18 or older | 830 | NR | PHQ-9 (10) |
| Woldetensay | 2018 | PLoS One | 3.2 | Ethiopia | Pregnant women | 246 | 28 | PHQ-9 (8) |
| Xia | 2020 | Epilepsy Behav | 2.9 | China | Patients with epilepsy aged 18 and older | 213 | 35 | NDDI-E (12) |
| Xia | 2019 | Epilepsy Behav | 2.9 | China | Adult outpatients with epilepsy | 213 | 35 | PHQ-9 (6) |
| Yang | 2018 | Psychol Asses | 5.1 | China | Adolescents (grades 7-12) | 612 | 44 | CES-D (30) |
| Yardeni | 2020 | Psychooncology | 3.9 | Israel | Patients with cancer aged 7 to 21 years | 91 | 11 | PROMIS-D (13) |
| Ye | 2020 | BMJ Open | 2.7 | China | Outpatients and inpatients with psoriasis | 148 | 30 | PHQ-9 (9) |
| Yuan | 2019 | J Psychosom Res | 3 | China | Inpatients with acute coronary syndrome | 782 | 122 | PHQ-9 (10) |
| Zachar-Tirado | 2021 | J Head Trauma Rehabil | 2.7 | United States | Outpatients with traumatic brain injury aged 12 to 17 years | 101 | 42 | PHQ-A (5) |
| Zinchuk | 2020 | Epilepsy Behav | 2.9 | Russia | Adult inpatients and outpatients with epilepsy | 175 | 76 | NDDI-E (13) |

Abbreviations: AViDI-18 = Akena Visual Depression Inventory – 18; ADAMS – Depressive Mood Subscale = Anxiety, Depression And Mood Scale; A-CSDD = Arabic version of the Cornell Scale for Depression in Dementia; BDI-I = Beck Depression Inventory-I; BDI-II = Beck Depression Inventory-II; BDI = Beck Depression Inventory; BEDS = Brief Edinburgh Depression Scale; CESD-R = Center for Epidemiologic Studies Depression Scale; CES-D = Center for Epidemiologic Studies Depression Scale; CESD-R = Center for Epidemiological Studies Depression Scale – Revised; CDST = Children Depression Screening Tool; CIDT-MD = Community Informant Detection Tool for Maternal Depression; CD-RISC 10 = Connor-Davidson Resilience Scale-10; DSSS-DS = Depression and Somatic; Symptoms Scale – Depression Subscale (DS); DI-MHD = Depression Inventory for Maintenance Hemodialysis Patients; DRQ-7 = Depression Risk Questionnaire 7; DT = Distress Thermometer; EPDS-3 = Edinburgh Postnatal Depression Scale-3; EPDS = Edinburgh Postnatal Depression Scale; GDS-15 = Geriatric Depression Scale-15; GDS-4 = Geriatric Depression Scale-4; GDS-5 = Geriatric Depression Scale-5; GDS = Geriatric Depression Scale; HSCL-25 = Hopkins Symptom Checklist-25; HSCL = Hopkins Symptom Checklist; HADS-D = Hospital Anxiety and Depression Scale – Depression subscale; HADS = Hospital Anxiety and Depression Scale; HIV = Human Immunodeficiency Virus; K10 = Kessler Psychological Distress Scale-10; MGMQ = Matthey Generic Mood Questionnaire; MFQ = Mood and Feeling Questionnaire; N/A = Not Applicable; NDDI-E = Neurologic Depression Disorders Inventory in Epilepsy; NR = Not Reported; PHQ-2 = Patient Health Questionnaire-2; PHQ-9 = Patient Health Questionnaire-9; PROMIS-D = Patient Reported Outcomes Measurement Information System – Depression module; PROMIS-D-CAT = Patient Reported Outcomes Measurement Information System Depression Computer Adaptive Test; PROMIS-D-SF = Patient Reported Outcomes Measurement Information System Depression Short Form; DePreS = Post-stroke Depression Prediction Scale; PDPI-R = Postpartum Depression Predictors Inventory-Revised; PDSS = Postpartum Depression Screening Scale; RCADS-D = Revised Children’s Anxiety and Depression – Depression module; SDS = Self-Rating Depression Scale; WMH-ICS = World Mental Health-International College Student; ZLDSI = Zanmi Lasante Depression Symptom Inventory.

**S3 Appendix. Sample size of primary studies of the diagnostic accuracy of depression screening tools**

| **First author, last name** | **Year** | **Journal** | **2020 impact factor** | **Country(ies)** | **Population** | ***N* screened and  interviewed** | ***N* cases** | **Sample size  calculation?** | **Appropriate method?** | **Why inappropriate method?** |
| --- | --- | --- | --- | --- | --- | --- | --- | --- | --- | --- |
| Akena | 2018 | Br J Psychiatry | 9.3 | Uganda; South Africa | Patients in low literacy settings | 343 | 78 | Yes | Yes | N/A |
| Alves | 2019 | Midwifery | 2.4 | Portugal | Perinatal women | 140 | 6 | No | N/A | N/A |
| Arturo Cassiani-Miranda | 2021 | Gen Hosp Psychiatry | 3.2 | Colombia | Adult primary care patients | 243 | 51 | Yes | No | Used formula designed to compare a difference in two proportions (e.g., sensitivity for test 1 versus test 2), which was not relevant for their study, since they assessed the accuracy of one test without reference to another. |
| Aslan | 2020 | Front Psychiatry | 4.2 | Chile | Self-dependent primary care outpatients aged 65 to 80 | 577 | 21 | No | N/A | N/A |
| Baldellou Lopez | 2021 | J Psychosom Res | 3 | United Kingdom | Adults with dissociative seizures in the previous 8 weeks | 368 | 114 | No | N/A | N/A |
| Ballester | 2019 | PLoS One | 3.2 | Spain | First year university students aged 18 to 24 | 287 | 26 | No | N/A | N/A |
| Baumgartner | 2019 | Psychiatr Prax | 1.5 | Austria | Individuals aged 18 to 65 from the general population | 504 | 39 | No | N/A | N/A |
| Bautovich | 2018 | Australas Psychiatry | 1.4 | Australia | Patients aged over 18 years with chronic haemodialysis | 45 | 6 | No | N/A | N/A |
| Bernstein | 2018 | Inflamm Bowel Dis | 5.3 | United States | Individuals with inflammatory bowel disease aged 18 years or older | 242 | 21 | Yes | No | Provided some assumptions for a sample size calculation (sensitivity, specificity, precision, alpha) but not all necessary assumptions to calculate (proportion of cases and non-cases). |
| Bhana | 2019 | S Afr Med J | 1.6 | South Africa | Outpatients aged 18 years or older attending primary healthcare clinics | 1206 | 57 | Yes | Yes | N/A |
| Binagwaho | 2021 | BMC Pediatr | 2.1 | Rwanda | Children with HIV aged 7 to 14 | 296 | 42 | Yes | No | Listed some assumptions but not all necessary assumptions and it was not clear what formula was used, or how it was calculated. |
| Blanco | 2019 | Aging Ment Health | 3.7 | Spain | Non-professional caregivers | 294 | 30 | No | N/A | N/A |
| Borghero | 2018 | Rev Med Chil | 0.6 | Chile | Adolescents aged 15 to 19 years | 245 | 210 | Yes | No | Provided some assumptions but no target for power (e.g., precision of intervals or test between groups) |
| Butnoriene | 2018 | BMC Psychiatry | 3.6 | Lithuania | Individuals aged 45 years or older with and without metabolic syndrome | 1115 | 201 | No | N/A | N/A |
| Cassiani-Miranda | 2021 | Rev Colomb Psiquiatr | NA | Colombia | Primary care patients aged 18 to 65 years | 243 | 53 | Yes | Yes | N/A |
| Chenneville | 2019 | J Affect Disord | 4.8 | United States | People with HIV aged 12 to 25 years receiving care in an integrated care setting | 121 | 37 | No | N/A | N/A |
| Clover | 2018 | Qual Life Res | 4.1 | Australia | Outpatients with cancer | 132 | 18 | No | N/A | N/A |
| Colomo | 2021 | Arch Bronconeumol | NA | Spain | Adult patients with bronchiectasis | 52 | 18 | No | N/A | N/A |
| Cruzado | 2018 | Support Care Cancer | 3.6 | Spain | Adult cancer patients | 130 | 6 | No | N/A | N/A |
| Cumbe | 2020 | BMC Psychiatry | 3.6 | Mozambique | Prenatal, postnatal, and general outpatients | 502 | 43 | No | N/A | N/A |
| Dajpratham | 2020 | BMC Psychiatry | 3.6 | Thailand | First-time stroke patients aged 45 and older | 115 | 23 | No | N/A | N/A |
| Degefa | 2020 | BMC Psychiatry | 3.6 | Ethiopia | Adult outpatients with cancer | 163 | 25 | Yes | Yes | N/A |
| Durmaz | 2018 | North Clin Istanb | NA | Turkey | Older adults (aged 65 or over) outpatients visiting a geriatric clinic | 329 | 81 | Yes | Yes | N/A |
| Eriksen | 2019 | J Affect Disord | 4.8 | Norway | Home-dwelling adults aged 60 years or older | 194 | 56 | No | N/A | N/A |
| Figueiredo-Duarte | 2019 | Aging Ment Health | 3.7 | Portugal | Institutionalized adults aged 60 or older | 186 | 20 | No | N/A | N/A |
| Fuseekul | 2021 | Child Adolesc Psychiatry Ment Health | 3 | Thailand | Secondary school students aged 12-18 | 103 | 31 | No | N/A | N/A |
| Gallis | 2018 | PeerJ | 3 | Pakistan | Community-based pregnant women | 1731 | 454 | Yes | No | Indicated that sample size was based on a desired effect size, which was not reported, with no other information. |
| Getting it Right  Collaborative Group | 2019 | Med J Aust | 7.7 | Australia | Adults (18 years or older) identifying as Indigenous Australians | 500 | 108 | Yes | Yes | N/A |
| Ghazisaeedi | 2021 | Int J Ment Health Addict | 3.8 | Iran | Undergraduate medical students | 400 | NR | No | N/A | N/A |
| Gholizadeh | 2019 | Contemp Nurse | 1.8 | Iran | Coronary artery disease inpatients aged 20 or above | 150 | 33 | Yes | No | Method based on having 10 participants per questionnaire item with no other explanation. |
| Gouweloos-Trines | 2019 | Eur J Psychotraumatol | 4.1 | Netherlands | Survivors of an airplane crash aged 14 years or older | 38 | 6 | No | N/A | N/A |
| Green | 2018 | J Affect Disord | 4.8 | Kenya | Perinatal women (pregnant and postpartum) | 192 | 10 | No | N/A | N/A |
| Guerin | 2018 | J Affect Disord | 4.8 | United States | Adults aged 18 to 54 versus 55 to 80 | 311 | 117 | No | N/A | N/A |
| Hamers | 2018 | J Appl Res Intellect Disabil | 2.7 | Netherlands | People with intellectual disabilities aged 18 to 49 years | 43 | 8 | Yes | No | Stated a number targeted for measure reliability analyses with no other justification and no target sample size for accuracy. |
| Herizchi | 2020 | Health Promot Perspect | 0.6 | Iran | Adults 60 years and older | 387 | 44 | No | N/A | N/A |
| Hirt | 2020 | J Affect Disord | 4.8 | Netherlands; Germany | Hospitalized patients with a diagnosis of ischemic stroke or intracerebral hemorrhage | 93 | 17 | Yes | No | Authors cited a viable sample size calculation method, but the number they reported was a small fraction of number needed based on that method. |
| Hitchon | 2020 | Arthritis Care Res | 4.8 | Canada | Outpatients with rheumatoid arthritis aged 18 or older | 150 | 17 | No | N/A | N/A |
| Housen | 2018 | Transcult Psychiatry | 2.2 | India | Adults general medical outpatients aged 18 years or older | 290 | 81 | No | N/A | N/A |
| Indu | 2018 | Asian J Psychiatr | 3.5 | India | Women aged 18-60 years in primary care | 228 | 57 | Yes | Yes | N/A |
| Jahn | 2018 | Psychiatr Prax | 1.5 | Austria | Adults from the general population | 508 | 37 | No | N/A | N/A |
| Jokelainen | 2019 | Scand J Prim Health Care | 2.6 | Finland | Older adults born in 1935 | 505 | 24 | No | N/A | N/A |
| Kagee | 2020 | Gen Hosp Psychiatry | 3.2 | South Africa | Patients receiving outpatient HIV care | 688 | 170 | No | N/A | N/A |
| Karam | 2018 | Alzheimers Dement  (Amst) | 21.6 | Lebanon | Individuals aged between 63 and 101 years | 56 | 10 | No | N/A | N/A |
| Kim | 2020 | Seizure | 3.2 | Korea | Outpatients aged 18 or older with epilepsy | 213 | 48 | No | N/A | N/A |
| Kokoszka | 2020 | Prim Care Diabetes | 2.5 | Poland | Outpatients and inpatients with Type 2 diabetes aged 18 or older | 101 | 35 | Yes | No | Method based on power to determine that the test was more accurate than chance based on AUC. |
| Kwan | 2019 | Semin Arthritis Rheum | 5.5 | Canada | Adult outpatients with systemic lupus erythematosus | 159 | 23 | No | N/A | N/A |
| Kyranou | 2020 | BMC Psychiatry | 3.6 | Greece | Outpatients receiving chemotherapy for solid tumours | 152 | NR | No | N/A | N/A |
| Lafont | 2021 | Oncologist | 5 | France | Inpatients and outpatients aged 70 or older with solid or hematological cancers | 830 | 208 | No | N/A | N/A |
| Legha | 2020 | Confl Health | 2.7 | Haiti | Transitional age youth (18-22) attending schools | 120 | 9 | No | N/A | N/A |
| Liu | 2019 | Neuropsychiatr Dis Treat | 2.6 | Taiwan | Outpatients with chronic low back pain | 225 | 21 | No | N/A | N/A |
| Loades | 2020 | Eur Child Adolesc Psychiatry | 4.8 | England | Adolescents (12-18 years) with Chronic Fatigue Syndrome/Myalgic Encephalomyelitis | 164 | 33 | No | N/A | N/A |
| Lydsdottir | 2019 | Midwifery | 2.4 | Iceland | Pregnant women | 474 | NR | No | N/A | N/A |
| Macêdo | 2018 | Lupus | 2.9 | Brazil | Patients with systemic lupus erythematosus | 108 | 21 | No | N/A | N/A |
| Marrie | 2018 | Mult Scler Relat Disord | 4.3 | Canada | Outpatients with multiple sclerosis aged 18 years or older, attending a multiple sclerosis clinic | 253 | 26 | Yes | No | Provided some assumptions for a sample size calculation (sensitivity, specificity, precision, alpha) but not all necessary assumptions to calculate (proportion of cases and non-cases). |
| Martínez | 2020 | J Clin Psychol | 2.9 | Chile | Mothers 18 years and older with infants aged 2-6 months | 298 | 63 | Yes | No | Method was not relevant for establishing precision of accuracy estimates. |
| Massai | 2018 | Parkinsons Dis | 2.7 | Italy | Patients with Parkinson's disease | 74 | 23 | No | N/A | N/A |
| Matthey | 2019 | J Affect Disord | 4.8 | Australia | Pregnant women attending antenatal clinic | 247 | 7 | No | N/A | N/A |
| McCartney | 2020 | Epilepsy Behav | 2.9 | Australia | Adult inpatients aged 18 to 77 years with epilepsy or psychogenic non-epileptic seizure | 485 | 115 | No | N/A | N/A |
| Mohsin | 2021 | Int J Environ Res Public Health | 3.4 | Pakistan | Pregnant women and mothers with children aged 3 years or less | 425 | 100 | Yes | Yes | N/A |
| Molebatsi | 2020 | BMC Psychiatry | 3.6 | Botswana | Adult primary care patients | 257 | 105 | No | N/A | N/A |
| Muramatsu | 2018 | Gen Hosp Psychiatry | 3.2 | Japan | Primary care patients | 284 | 93 | No | N/A | N/A |
| Nabbe | 2019 | PLoS One | 3.2 | France | General practice patients aged 18 or older | 142 | 73 | Yes | Yes | N/A |
| Navarrete | 2019 | Salud Publica Mex | 2 | Mexico | Women during  pregnancy | 210 | NR | No | N/A | N/A |
| Park | 2020 | Front Psychol | 3 | Korea | Adults recruited online and during visit to hospital (patients, visitors, staff) | 1145 | 96 | No | N/A | N/A |
| Peng | 2020 | Dermatol Ther | 2.9 | China | Outpatients with acne aged 18 to 24 years | 258 | 47 | No | N/A | N/A |
| Rancans | 2018 | Ann Gen Psychiatry | 3.5 | Latvia | Primary care outpatients aged 18 years or older | 1467 | 150 | No | N/A | N/A |
| Rashid | 2021 | Epilepsy Behav | 2.9 | India | Adult outpatients with epilepsy | 449 | 180 | No | N/A | N/A |
| Rashid | 2019 | Epilepsy Behav | 2.9 | India | Outpatients with epilepsy aged 18 or older | 217 | 90 | No | N/A | N/A |
| Recklitis | 2020 | Cancer | 6.9 | United States | Adult cancer survivors aged 18 to 40 years | 249 | 32 | No | N/A | N/A |
| Risal | 2019 | J Nepal Health Res Counc | NA | Nepal | Adult outpatients aged 60 years or older | 106 | 51 | No | N/A | N/A |
| Rodríguez-Mayoral | 2018 | Palliat Support Care | 2.3 | Mexico | Outpatients aged 18 and older with advanced cancer receiving palliative care | 70 | 14 | Yes | No | Method based on having 10 participants per questionnaire item with no furhter explanation. |
| Saal | 2018 | AIDS Care | 2.3 | South Africa | Individuals aged 18 years or older seeking HIV testing | 500 | 72 | No | N/A | N/A |
| Saldivia | 2019 | Rev Med Chil | 0.6 | Chile | Primary care patients aged 18 to 75 years | 1738 | 187 | No | N/A | N/A |
| Sampasa-Kanyinga | 2018 | PLoS One | 3.2 | Canada | Military personnel | 6700 | 208 | Yes | No | Method based on desired precision around disorder prevalence but not related to test accuracy. |
| Sasaki | 2019 | J Obstet Gynaecol Res | 1.7 | Japan | Women 4 days to 1 month postpartum | 80 | 9 | Yes | No | Method based on power to determine that the test was more accurate than chance based on AUC. |
| Scoppetta | 2021 | J Affect Disord | 4.8 | Colombia | Adult primary care patients | 243 | 52 | Yes | No | Indicated that method based on comparing new test to gold standard, but no assumptions or method provided. |
| Searle | 2019 | Assessment | 4.7 | Regular-serving military personnel | Australia | 1730 | 74 | No | N/A | N/A |
| Shaheen | 2019 | Am J Mens Health | 2.8 | Saudi Arabia | Fathers of newborns aged up to 6 months | 57 | 9 | No | N/A | N/A |
| Shih | 2020 | Seizure | 3.2 | Taiwan | Inpatients and outpatients with epilepsy aged 20 or older | 109 | 20 | No | N/A | N/A |
| Silagadze | 2019 | Epilepsy Behav | 2.9 | Georgia | Adult outpatients with epilepsy | 130 | 31 | No | N/A | N/A |
| Smith Fawzi | 2019 | Neurol Psychiatry Brain Res | NA | Tanzania | Primary care patients aged 18 years and older | 174 | 18 | No | N/A | N/A |
| Smith-Nielsen | 2018 | BMC Psychiatry | 3.6 | Denmark | Women aged 18 or older who were 2 to 10 months postpartum | 324 | 118 | No | N/A | N/A |
| Tan | 2018 | J Am Psychiatr Nurses Assoc | 2.4 | Singapore | Adult outpatients aged 65 years or older attending a general geriatric outpatient clinic | 77 | 6 | Yes | Yes | N/A |
| Tran | 2019 | J Affect Disord | 4.8 | Indonesia | Adolescents (aged between 16 and 18 years) attending senior schools | 189 | NR | No | N/A | N/A |
| Tschorn | 2019 | Psychiatr Prax | 1.5 | Germany | Patients with coronary heart disease | 1019 | 83 | No | N/A | N/A |
| Udedi | 2019 | BCM Psychiatry | 3.6 | Malawi | Outpatients with type-2 diabetes | 323 | 58 | Yes | Yes | N/A |
| Urtasun | 2019 | BMC Psychiatry | 3.6 | Argentina | Adults attending primary care or mental health clinics | 169 | 102 | Yes | Yes | N/A |
| van Heyningen | 2019 | Glob Ment Health | 3.5 | South Africa | Pregnant women aged 18 years or older receiving outpatient antenatal care | 376 | 81 | No | N/A | N/A |
| van Heyningen | 2018 | PLos One | 3.2 | South Africa | Pregnant women attending a primary care antenatal clinic | 376 | 81 | No | N/A | N/A |
| Vázquez | 2019 | J Affect Disord | 4.8 | Spain | Pregnant women | 569 | 40 | No | N/A | N/A |
| Vogeli | 2018 | Res Nurs Health | 2.2 | United States | Mothers of 4- to 15-month-old infants | 238 | 36 | No | N/A | N/A |
| Vrublevska | 2018 | Nord J Psychiatry | 2.2 | Latvia | Adult (aged 18 years or older) primary care patients | 272 | 37 | No | N/A | N/A |
| Wang | 2019 | Ther Apher Dial | 1.8 | China | Patients aged 18 years or older undergoing maintenance hemodialysis | 112 | 24 | No | N/A | N/A |
| Wang | 2018 | J Geriatr Psychiatry Neurol | 2.7 | United States | Older adult outpatients with ischemic heart disease and prior stroke | 147 | 35 | No | N/A | N/A |
| Weihs | 2018 | Psychooncology | 3.9 | United States | Patients with breast cancer | 82 | 14 | No | N/A | N/A |
| Williams | 2021 | Assessment | 4.7 | United States | Adults with autism spectrum disorder aged 18 to 45 years | 66 | 24 | No | N/A | N/A |
| Williams | 2020 | Neurol Clin Pract | NA | Canada | Outpatients with neurologic disorders aged 18 or older | 830 | NR | No | N/A | N/A |
| Woldetensay | 2018 | PLoS One | 3.2 | Ethiopia | Pregnant women | 246 | 28 | No | N/A | N/A |
| Xia | 2020 | Epilepsy Behav | 2.9 | China | Patients with epilepsy aged 18 and older | 213 | 35 | No | N/A | N/A |
| Xia | 2019 | Epilepsy Behav | 2.9 | China | Adult outpatients with epilepsy | 213 | 35 | No | N/A | N/A |
| Yang | 2018 | Psychol Asses | 5.1 | China | Adolescents (grades 7-12) | 612 | 44 | No | N/A | N/A |
| Yardeni | 2020 | Psychooncology | 3.9 | Israel | Patients with cancer aged 7 to 21 years | 91 | 11 | No | N/A | N/A |
| Ye | 2020 | BMJ Open | 2.7 | China | Outpatients and inpatients with psoriasis | 148 | 30 | No | N/A | N/A |
| Yuan | 2019 | J Psychosom Res | 3 | China | Inpatients with acute coronary syndrome | 782 | 122 | No | N/A | N/A |
| Zachar-Tirado | 2021 | J Head Trauma Rehabil | 2.7 | United States | Outpatients with traumatic brain injury aged 12 to 17 years | 101 | 42 | No | N/A | N/A |
| Zinchuk | 2020 | Epilepsy Behav | 2.9 | Russia | Adult inpatients and outpatients with epilepsy | 175 | 76 | No | N/A | N/A |

Abbreviations: AUC = Area under the ROC Curve; AViDI-18 = Akena Visual Depression Inventory – 18; ADAMS – Depressive Mood Subscale = Anxiety, Depression And Mood Scale; A-CSDD = Arabic version of the Cornell Scale for Depression in Dementia; BDI-I = Beck Depression Inventory-I; BDI-II = Beck Depression Inventory-II; BDI = Beck Depression Inventory; BEDS = Brief Edinburgh Depression Scale; CESD-R = Center for Epidemiologic Studies Depression Scale; CES-D = Center for Epidemiologic Studies Depression Scale; CESD-R = Center for Epidemiological Studies Depression Scale – Revised; CDST = Children Depression Screening Tool; CI = Confidence Interval; CIDT-MD = Community Informant Detection Tool for Maternal Depression; CD-RISC 10 = Connor-Davidson Resilience Scale-10; DSSS-DS = Depression and Somatic; Symptoms Scale – Depression Subscale (DS); DI-MHD = Depression Inventory for Maintenance Hemodialysis Patients; DRQ-7 = Depression Risk Questionnaire 7; DT = Distress Thermometer; EPDS-3 = Edinburgh Postnatal Depression Scale-3; EPDS = Edinburgh Postnatal Depression Scale; GDS-15 = Geriatric Depression Scale-15; GDS-4 = Geriatric Depression Scale-4; GDS-5 = Geriatric Depression Scale-5; GDS = Geriatric Depression Scale; HSCL-25 = Hopkins Symptom Checklist-25; HSCL = Hopkins Symptom Checklist; HADS-D = Hospital Anxiety and Depression Scale – Depression subscale; HADS = Hospital Anxiety and Depression Scale; HIV = Human Immunodeficiency Virus; K10 = Kessler Psychological Distress Scale-10; MGMQ = Matthey Generic Mood Questionnaire; MFQ = Mood and Feeling Questionnaire; N/A = Not Applicable; NDDI-E = Neurologic Depression Disorders Inventory in Epilepsy; NR = Not Reported; PHQ-2 = Patient Health Questionnaire-2; PHQ-9 = Patient Health Questionnaire-9; PROMIS-D = Patient Reported Outcomes Measurement Information System – Depression module; PROMIS-D-CAT = Patient Reported Outcomes Measurement Information System Depression Computer Adaptive Test; PROMIS-D-SF = Patient Reported Outcomes Measurement Information System Depression Short Form; DePreS = Post-stroke Depression Prediction Scale; PDPI-R = Postpartum Depression Predictors Inventory-Revised; PDSS = Postpartum Depression Screening Scale; RCADS-D = Revised Children’s Anxiety and Depression – Depression module; SDS = Self-Rating Depression Scale; WMH-ICS = World Mental Health-International College Student; ZLDSI = Zanmi Lasante Depression Symptom Inventory.

**S4 Appendix: Confidence intervals of primary studies of the diagnostic accuracy of depression screening tools**

| **First author, last name** | **Year** | **Journal** | **2020 impact factor** | ***N* Screened and  interviewed** | ***N* cases** | **Screening tool and cutoff threshold used for precision estimates** | **Sensitivity (95% CI)** | **CI width** | **Specificity (95% CI)** | **CI width** | **CIs provided in  publication?** | **CIs calculated?** | **CI calculation reason?** |
| --- | --- | --- | --- | --- | --- | --- | --- | --- | --- | --- | --- | --- | --- |
| Akena | 2018 | Br J Psychiatry | 9.3 | 343 | 78 | AViDI-18 (16) | 87.2 (78.0 to 92.9) | 14.9 | 84.9 (80.1 to 88.7) | 8.6 | No | Yes | Not Provided |
| Alves | 2019 | Midwifery | 2.4 | 140 | 6 | PDPI-R (4.5) | 83.3 (43.6 to 97.0) | 53.3 | 85.8 (78.9 to 90.7) | 11.8 | No | Yes | Not Provided |
| Arturo Cassiani-Miranda | 2021 | Gen Hosp Psychiatry | 3.2 | 243 | 51 | HADS-D (4) | 78.4 (65.4 to 87.5) | 22.1 | 74.0 (67.3 to 79.7) | 12.3 | No | Yes | Not Provided |
| Aslan | 2020 | Front Psychiatry | 4.2 | 577 | 21 | PHQ-9 (6) | 95.2 (77.3 to 99.2) | 21.8 | 76.1 (72.4 to 79.4) | 7.1 | No | Yes | Not Provided |
| Baldellou Lopez | 2021 | J Psychosom Res | 3 | 368 | 114 | PHQ-9 (13) | 80.7 (72.5 to 86.9) | 14.4 | 66.9 (60.9 to 72.4) | 11.5 | No | Yes | Not Provided |
| Ballester | 2019 | PLoS One | 3.2 | 287 | 26 | WMH-ICS online screening scale (15) | 69.2 (50.0 to 83.5) | 33.5 | 89.7 (85.4 to 92.8) | 7.4 | No | Yes | Not Provided |
| Baumgartner | 2019 | Psychiatr Prax | 1.5 | 504 | 39 | GDS-15 (2/3) | 71.8 (57.6 to 86.0) | 28.4 | 82.4 (78.9 to 85.9) | 7.0 | Yes | No | N/A |
| Bautovich | 2018 | Australas Psychiatry | 1.4 | 45 | 6 | BDI (18) | 100.0 (61.0 to 100.0) | 39.0 | 89.7 (76.4 to 95.9) | 19.5 | No | Yes | Not Provided |
| Bernstein | 2018 | Inflamm Bowel Dis | 5.3 | 242 | 21 | PHQ-2 (3) | 81.0 (58.0 to 95.0) | 37.0 | 88.0 (82.0 to 92.0) | 10.0 | Yes | No | N/A |
| Bhana | 2019 | S Afr Med J | 1.6 | 1206 | 57 | PHQ-2 (3) | 57.9 (45.0 to 69.8) | 24.8 | 77.4 (74.9 to 79.7) | 4.8 | No | Yes | Not Provided |
| Binagwaho | 2021 | BMC Pediatr | 2.1 | 296 | 42 | CDST (6) | 88.1 (75.0 to 94.8) | 19.8 | 96.5 (93.4 to 98.1) | 4.7 | No | Yes | Not Provided |
| Blanco | 2019 | Aging Ment Health | 3.7 | 294 | 30 | CD-RISC 10 (23) | 70.0 (52.1 to 83.3) | 31.2 | 68.2 (62.3 to 73.5) | 11.2 | No | Yes | Not Provided |
| Borghero | 2018 | Rev Med Chil | 0.6 | 245 | 210 | PHQ-9 (11) | 86.2 (80.9 to 90.2) | 9.3 | 82.9 (67.3 to 91.9) | 24.6 | No | Yes | Not Provided |
| Butnoriene | 2018 | BMC Psychiatry | 3.6 | 1115 | 201 | HADS-D (8) | 87.0 (81.0 to 91.0) | 10.0 | 74 .0 (71.0 to 77.0) | 6.0 | Yes | No | N/A |
| Cassiani-Miranda | 2021 | Rev Colomb Psiquiatr | NA | 243 | 53 | PHQ-9 (7) | 90.4 (81.4 to 99.4) | 18.0 | 81.68 (75.9 to 87.4) | 11.5 | Yes | No | N/A |
| Chenneville | 2019 | J Affect Disord | 4.8 | 121 | 37 | PHQ-9 (5.5) | 78.4 (62.8 to 88.6) | 25.8 | 73.8 (63.5 to 82.0) | 18.5 | No | Yes | Not Provided |
| Clover | 2018 | Qual Life Res | 4.1 | 132 | 18 | PROMIS-D-CAT (53) | 83.0 (67.0 to 100.0) | 33.0 | 70.0 (62.0 to 78.0) | 16.0 | Yes | No | N/A |
| Colomo | 2021 | Arch Bronconeumol | NA | 52 | 18 | HADS-D (9) | 83.3 (60.8 to 94.2) | 33.4 | 97.1 (85.1 to 99.5) | 14.4 | No | Yes | Not Provided |
| Cruzado | 2018 | Support Care Cancer | 3.6 | 130 | 6 | HADS-T (10) | 80.0 (68.0 to 93.0) | 25.0 | 92.0 (86.0 to 97.0) | 11.0 | Yes | No | N/A |
| Cumbe | 2020 | BMC Psychiatry | 3.6 | 502 | 43 | PHQ-9 (9) | 46.5 (32.5 to 61.1) | 28.6 | 93.5 (90.8 to 95.4) | 4.6 | No | Yes | Not Provided |
| Dajpratham | 2020 | BMC Psychiatry | 3.6 | 115 | 23 | PHQ-9 (6) | 87.0 (66.4 to 97.2) | 30.8 | 75.0 (64.9 to 83.4) | 18.5 | Yes | No | N/A |
| Degefa | 2020 | BMC Psychiatry | 3.6 | 163 | 25 | PHQ-9 (4) | 88.0 (70.0 to 95.8) | 25.8 | 78.3 (70.7 to 84.3) | 13.7 | No | Yes | Not Provided |
| Durmaz | 2018 | North Clin Istanb | NA | 329 | 81 | GDS-15 (5) | 92.6 (84.8 to 96.6) | 11.8 | 91.1 (86.9 to 94.1) | 7.1 | No | Yes | Not Provided |
| Eriksen | 2019 | J Affect Disord | 4.8 | 194 | 56 | GDS-5 (2) | 73.2 (60.4 to 83.0) | 22.6 | 73.2 (65.2 to 79.9) | 14.6 | No | Yes | Not Provided |
| Figueiredo-Duarte | 2019 | Aging Ment Health | 3.7 | 186 | 20 | GDS (5/6) | 80.0 (58.4 to 91.9) | 33.5 | 77.1 (70.1 to 82.8) | 12.7 | No | Yes | Not Provided |
| Fuseekul | 2021 | Child Adolesc Psychiatry Ment Health | 3 | 103 | 31 | MFQ (28) | 96.8 (83.8 to 99.4) | 15.6 | 83.3 (73.1 to 90.2) | 17.1 | No | Yes | Not Provided |
| Gallis | 2018 | PeerJ | 3 | 1731 | 454 | PHQ-9 (10) | 94.7 (92.2 to 96.6) | 4.4 | 88.9 (87.0 to 90.6) | 3.6 | Yes | No | N/A |
| Getting it Right  Collaborative Group | 2019 | Med J Aust | 7.7 | 500 | 108 | PHQ-9 (10) | 84.0 (74 .0 to 91.0) | 17.0 | 77.0 (71.0 to 83.0) | 12.0 | Yes | No | N/A |
| Ghazisaeedi | 2021 | Int J Ment Health Addict | 3.8 | 400 | NR | PHQ-9 (5) | N/A | N/A | N/A | N/A | No | No | N/A |
| Gholizadeh | 2019 | Contemp Nurse | 1.8 | 150 | 33 | PHQ-9 (7) | 75.8 (59.0 to 87.2) | 28.2 | 76.9 (68.5 to 83.6) | 15.1 | No | Yes | Not Provided |
| Gouweloos-Trines | 2019 | Eur J Psychotraumatol | 4.1 | 38 | 6 | PHQ-2 (3) | 33.3 (9.7 to 70.0) | 60.3 | 90.6 (75.8 to 96.8) | 21.0 | No | Yes | Not Provided |
| Green | 2018 | J Affect Disord | 4.8 | 192 | 10 | EPDS (16) | 70.0 (39.7 to 89.2) | 49.5 | 72.0 (65.1 to 78.0) | 12.9 | No | Yes | Not Provided |
| Guerin | 2018 | J Affect Disord | 4.8 | 311 | 117 | GDS (5) | 71.8 (63.0 to 79.2) | 16.1 | 96.9 (93.4 to 98.6) | 5.2 | No | Yes | Not Provided |
| Hamers | 2018 | J Appl Res Intellect Disabil | 2.7 | 43 | 8 | ADAMS -  Depressive Mood Subscale (14) | 88.0 (53.0 to 98.0) | 45.0 | 80.0 (64.0 to 90.0) | 26.0 | Yes | No | N/A |
| Herizchi | 2020 | Health Promot Perspect | 0.6 | 387 | 44 | GDS (6) | 90.9 (78.8 to 96.4) | 17.6 | 73.4 (70.7 to 79.7) | 9.0 | Yes | No | N/A |
| Hirt | 2020 | J Affect Disord | 4.8 | 93 | 17 | DePreS (0) | 65.0 (42.0 to 87.0) | 45.0 | 74.0 (64.0 to 84.0 | 20.0 | Yes | No | N/A |
| Hitchon | 2020 | Arthritis Care Res | 4.8 | 150 | 17 | PHQ-2 (3) | 88.0 (63.0 to 98.0) | 35.0 | 84.0 (77.0 to 90.0) | 13.0 | Yes | No | N/A |
| Housen | 2018 | Transcult Psychiatry | 2.2 | 290 | 81 | HSCL-Depression Scale (1.57) | 77.8 (67.6 to 85.5) | 17.9 | 77.0 (70.9 to 82.2) | 11.3 | No | Yes | Not Provided |
| Indu | 2018 | Asian J Psychiatr | 3.5 | 228 | 57 | PHQ-9 (9) | 82.5 (72.4 to 92.6) | 20.2 | 90.1 (84.5 to 95.6) | 11.1 | Yes | No | N/A |
| Jahn | 2018 | Psychiatr Prax | 1.5 | 508 | 37 | CES-D (9/10) | 81.1 (68.5 to 93.7) | 25.2 | 74.3 (70.4 to 78.3) | 7.9 | Yes | No | N/A |
| Jokelainen | 2019 | Scand J Prim Health Care | 2.6 | 505 | 24 | SDS (39) | 79.2 (57.8 to 92.9) | 35.1 | 72.2 (67.9 to 76.1) | 8.2 | Yes | No | N/A |
| Kagee | 2020 | Gen Hosp Psychiatry | 3.2 | 688 | 170 | CESD-R (27) | 81.2 (74.5 to 86.8) | 12.3 | 82.3 (78.8 to 85.5) | 6.7 | Yes | No | N/A |
| Karam | 2018 | Alzheimers Dement  (Amst) | 21.6 | 56 | 10 | A-CSDD (13) | 70.0 (39.7 to 89.2) | 49.5 | 91.3 (79.7 to 96.6) | 16.9 | No | Yes | Not Provided |
| Kim | 2020 | Seizure | 3.2 | 213 | 48 | PHQ-9 (9.5) | 91.7 (80.4 to 96.7) | 16.3 | 75.8 (68.7 to 81.7) | 13.0 | No | Yes | Not Provided |
| Kokoszka | 2020 | Prim Care Diabetes | 2.5 | 101 | 35 | BDI (13) | 88.6 (75.9 to 95.9) | 20.0 | 86.4 (79.7 to 90.3) | 10.6 | Yes | No | N/A |
| Kwan | 2019 | Semin Arthritis Rheum | 5.5 | 159 | 23 | CES-D (16) | 82.6 (62.9 to 93.0) | 30.2 | 83.1 (75.9 to 88.5) | 12.6 | No | Yes | Not Provided |
| Kyranou | 2020 | BMC Psychiatry | 3.6 | 152 | NR | DT (4) | 85.4 (71.6 to 93.1) | 21.6 | 60.4 (51.1 to 69.0) | 17.9 | No | Yes | Not Provided |
| Lafont | 2021 | Oncologist | 5 | 830 | 208 | GDS-4 (1) | 90.0 (87.5 to 92.2) | 4.7 | 89.0 (87.4 to 90.5) | 3.1 | Yes | No | N/A |
| Legha | 2020 | Confl Health | 2.7 | 120 | 9 | ZLDSI (12) | 100.0 (70.1 to 100.0) | 29.9 | 73.9 (65.0 to 81.1) | 16.1 | No | Yes | Not Provided |
| Liu | 2019 | Neuropsychiatr Dis Treat | 2.6 | 225 | 21 | DSSS-DS (15) | 100.0 (84.5 to 100.0) | 15.5 | 88.7 (83.7 to 92.4) | 8.7 | No | Yes | Not Provided |
| Loades | 2020 | Eur Child Adolesc Psychiatry | 4.8 | 164 | 33 | RCADS-D (14) | 90.9 (76.84 to 96.9) | 20.4 | 59.5 (51.0 to 67.6) | 16.6 | No | Yes | Not Provided |
| Lydsdottir | 2019 | Midwifery | 2.4 | 474 | NR | EPDS (11) | N/A | N/A | N/A | N/A | No | No | N/A |
| Macêdo | 2018 | Lupus | 2.9 | 108 | 21 | BDI (13) | 95.2 (77.3 to 99.2) | 21.8 | 85.1 (76.1 to 91.1) | 15.0 | No | Yes | Not Provided |
| Marrie | 2018 | Mult Scler Relat Disord | 4.3 | 253 | 26 | PHQ-9 (10) | 84.0 (64.0 to 95.0) | 31.0 | 76.0 (70.0 to 82.0) | 12.0 | Yes | No | N/A |
| Martínez | 2020 | J Clin Psychol | 2.9 | 298 | 63 | EPDS-3 (8/9) | 93.7 (84.8 to 97.5) | 12.7 | 87.2 (82.4 to 90.9) | 8.5 | No | Yes | Not Provided |
| Massai | 2018 | Parkinsons Dis | 2.7 | 74 | 23 | GDS (15-16) | 87.0 (67.9 to 95.5) | 27.6 | 82.4 (69.7 to 90.4) | 20.7 | No | Yes | Not Provided |
| Matthey | 2019 | J Affect Disord | 4.8 | 247 | 7 | MGMQ (Lower Bother Impact threshold) | 75.0 (58.9 to 86.2) | 27.3 | 80.1 (74.2 to 84.9) | 10.7 | No | Yes | Not Provided |
| McCartney | 2020 | Epilepsy Behav | 2.9 | 485 | 115 | HADS-D (7) | 70.4 (61.5 to 78.0) | 16.5 | 83.0 (78.8 to 86.5) | 7.7 | No | Yes | Not Provided |
| Mohsin | 2021 | Int J Environ Res Public Health | 3.4 | 425 | 100 | CIDT-MD (3.5) | 81.0 (72.2 to 87.5) | 15.3 | 69.5 (64.3 to 74.3) | 10.0 | No | Yes | Not Provided |
| Molebatsi | 2020 | BMC Psychiatry | 3.6 | 257 | 105 | PHQ-9 (9) | 72.4 (62.8 to 80.7) | 17.9 | 76.3 (68.7 to 82.8) | 14.1 | Yes | No | N/A |
| Muramatsu | 2018 | Gen Hosp Psychiatry | 3.2 | 284 | 93 | PHQ-9 (10) | 90.3 (82.6 to 94.8) | 12.2 | 76.4 (69.9 to 81.9) | 12.0 | No | Yes | Not Provided |
| Nabbe | 2019 | PLoS One | 3.2 | 142 | 73 | HSCL-25 (1.75) | 59.2 (43.6 to 80.9) | 37.3 | 91.4 (88.5 to 94.1) | 5.6 | Yes | No | N/A |
| Navarrete | 2019 | Salud Publica Mex | 2 | 210 | NR | Whooley questions (NR) | 94.7 (73.9 to 99.8) | 25.9 | 39.4 (32.4 to 46.8) | 14.4 | Yes | No | N/A |
| Park | 2020 | Front Psychol | 3 | 1145 | 96 | BDI-II (23) | 83.3 (74.6 to 89.5) | 14.8 | 86.8 (84.7 to 88.8) | 4.1 | No | Yes | Not Provided |
| Peng | 2020 | Dermatol Ther | 2.9 | 258 | 47 | PHQ-9 (9) | 95.7 (85.8 to 98.8) | 13.1 | 88.6 (83.6 to 92.2) | 8.6 | No | Yes | Not Provided |
| Rancans | 2018 | Ann Gen Psychiatry | 3.5 | 1467 | 150 | PHQ-9 (8) | 74.7 (64.1 to 83.0) | 18.9 | 85.7 (65.4 to 95.0) | 29.7 | No | Yes | Not Provided |
| Rashid | 2021 | Epilepsy Behav | 2.9 | 449 | 180 | NDDI-E (12) | 87.2 (81.6 to 91.3) | 9.8 | 90.7 (86.6 to 93.6) | 7.0 | No | Yes | Not Provided |
| Rashid | 2019 | Epilepsy Behav | 2.9 | 217 | 90 | NDDI-E (12) | 96.7 (90.7 to 98.9) | 8.2 | 84.3 (76.9 to 89.6) | 12.6 | No | Yes | Not Provided |
| Recklitis | 2020 | Cancer | 6.9 | 249 | 32 | PROMIS-D-SF (53.2) | 81.0 (63.0 to 92.0) | 29.0 | 74.0 (68.0 to 80.0) | 12.0 | Yes | No | N/A |
| Risal | 2019 | J Nepal Health Res Counc | NA | 106 | 51 | GDS-15 (5/6) | 86.3 (74.3 to 93.2) | 18.9 | 74.5 (61.7 to 84.2) | 22.5 | No | Yes | Not Provided |
| Rodríguez-Mayoral | 2018 | Palliat Support Care | 2.3 | 70 | 14 | BEDS (5) | 85.7 (60.1 to 96.0) | 35.9 | 62.5 (49.4 to 74.0) | 24.6 | No | Yes | Not Provided |
| Saal | 2018 | AIDS Care | 2.3 | 500 | 72 | BDI-I (20) | 66.7 (55.2 to 76.5) | 21.3 | 67.1 (62.5 to 71.3) | 8.9 | No | Yes | Not Provided |
| Saldivia | 2019 | Rev Med Chil | 0.6 | 1738 | 187 | PHQ-9 (7) | 80.2 (73.9 to 85.3) | 11.4 | 77.0 (74.8 to 79.0) | 4.2 | No | Yes | Not Provided |
| Sampasa-Kanyinga | 2018 | PLoS One | 3.2 | 6700 | 208 | K10 (10) | 86.9 (83.6 to 90.2) | 6.6 | 82.4 (81.4 to 83.4) | 2.0 | Yes | No | N/A |
| Sasaki | 2019 | J Obstet Gynaecol Res | 1.7 | 80 | 9 | EPDS (10) | 77.8 (45.3 to 93.7) | 48.4 | 77.5 (66.5 to 85.6) | 19.1 | No | Yes | Not Provided |
| Scoppetta | 2021 | J Affect Disord | 4.8 | 243 | 52 | PHQ-2 (2) | 87.0 (74.0 to 94.0) | 20.0 | 74.0 (66.0 to 79.0) | 13.0 | Yes | No | N/A |
| Searle | 2019 | Assessment | 4.7 | 1730 | 74 | K10 (19) | 86.5 (72.0 to 94.1) | 22.1 | 79.0 (77.0 to 80.9) | 3.9 | Yes | Yes | Incorrect |
| Shaheen | 2019 | Am J Mens Health | 2.8 | 57 | 9 | EPDS (8/9) | 77.8 (45.3 to 93.7) | 48.4 | 81.3 (68.1 to 89.8) | 21.7 | No | Yes | Not Provided |
| Shih | 2020 | Seizure | 3.2 | 109 | 20 | NDDI-E (15) | 85.0 (62.1 to 96.8) | 34.7 | 87.6 (79.0 to 93.7) | 14.7 | Yes | No | N/A |
| Silagadze | 2019 | Epilepsy Behav | 2.9 | 130 | 31 | NDDI-E (16) | 90.3 (75.1 to 96.7) | 21.6 | 93.9 (87.4 to 97.2) | 9.8 | No | Yes | Not Provided |
| Smith Fawzi | 2019 | Neurol Psychiatry Brain Res | NA | 174 | 18 | PHQ-9 (9) | 78.0 (52.0 to 94.0) | 42.0 | 87.0 (80.0 to 92.0) | 12.0 | Yes | No | N/A |
| Smith-Nielsen | 2018 | BMC Psychiatry | 3.6 | 324 | 118 | EPDS (11) | 79.2 (60.3 to 98.1) | 37.8 | 94.4 (90.5 to 98.3) | 7.8 | Yes | No | N/A |
| Tan | 2018 | J Am Psychiatr Nurses Assoc | 2.4 | 77 | 6 | Emoticon scale (5) | 0.0 (0.0 to 39.0) | 39.0 | 93.0 (84.6 to 97.0) | 12.4 | No | Yes | Not Provided |
| Tran | 2019 | J Affect Disord | 4.8 | 189 | NR | CESD-R (20) | N/A | N/A | N/A | N/A | No | No | N/A |
| Tschorn | 2019 | Psychiatr Prax | 1.5 | 1019 | 83 | HADS-D (7) | 83.1 (74.1 to 90.1) | 16.0 | 75.3 (72.5 to 78.0) | 5.5 | Yes | No | N/A |
| Udedi | 2019 | BCM Psychiatry | 3.6 | 323 | 58 | PHQ-9 (9) | 84.5 (73.1 to 91.6) | 18.5 | 81.9 (76.8 to 86.1) | 9.3 | No | Yes | Not Provided |
| Urtasun | 2019 | BMC Psychiatry | 3.6 | 169 | 102 | PHQ-9 (8) | 88.2 (80.6 to 93.1) | 12.6 | 86.6 (76.4 to 92.8) | 16.4 | No | Yes | Not Provided |
| van Heyningen | 2019 | Glob Ment Health | 3.5 | 376 | 81 | No name (1) | 95.1 (88.0 to 98.1) | 10.1 | 58.0 (52.3 to 63.5) | 11.2 | No | Yes | Not Provided |
| van Heyningen | 2018 | PLos One | 3.2 | 376 | 81 | EPDS (14) | 86.4 (77.3 to 92.2) | 14.9 | 81.0 (76.2 to 85.1) | 8.9 | No | Yes | Not Provided |
| Vázquez | 2019 | J Affect Disord | 4.8 | 569 | 40 | EPDS (9) | 67.5 (52.0 to 79.9) | 27.9 | 70.9 (66.9 to 74.6) | 7.7 | No | Yes | Not Provided |
| Vogeli | 2018 | Res Nurs Health | 2.2 | 238 | 36 | PDSS (80) | 75.0 (58.9 to 86.2) | 27.3 | 83.2 (77.4 to 87.7) | 10.3 | No | Yes | Not Provided |
| Vrublevska | 2018 | Nord J Psychiatry | 2.2 | 272 | 37 | PHQ-9 (10) | 86.5 (72.0 to 94.1) | 22.1 | 89.4 (84.8 to 92.7) | 7.9 | No | Yes | Not Provided |
| Wang | 2019 | Ther Apher Dial | 1.8 | 112 | 24 | DI-MHD (25) | 95.8 (79.8 to 99.3) | 19.5 | 86.4 (77.7 to 92.0) | 14.4 | No | Yes | Not Provided |
| Wang | 2018 | J Geriatr Psychiatry Neurol | 2.7 | 147 | 35 | CES-D (10) | 80.0 (63.0 to 91.0) | 28.0 | 71.0 (62.0 to 79.0) | 17.0 | Yes | No | N/A |
| Weihs | 2018 | Psychooncology | 3.9 | 82 | 14 | DRQ-7 (6) | 85.7 (60.1 to 96.0) | 35.9 | 73.5 (62.0 to 82.6) | 20.6 | No | Yes | Not Provided |
| Williams | 2021 | Assessment | 4.7 | 66 | 24 | BDI-II (14) | 62.5 (41.7 to 79.2] | 37.5 | 66.7 (52.4 to 81.0) | 28.6 | Yes | No | N/A |
| Williams | 2020 | Neurol Clin Pract | NA | 830 | NR | PHQ-9 (10) | 90.0 (81.0 to 97.0) | 16.0 | 85.0 (79.0 to 90.0) | 11.0 | Yes | No | N/A |
| Woldetensay | 2018 | PLoS One | 3.2 | 246 | 28 | PHQ-9 (8) | 82.1 (64.4 to 92.1) | 27.7 | 79.4 (73.5 to 84.2) | 10.7 | No | Yes | Not Provided |
| Xia | 2020 | Epilepsy Behav | 2.9 | 213 | 35 | NDDI-E (12) | 85.7 (70.6 to 93.7) | 23.1 | 79.8 (73.3 to 85.0) | 11.7 | No | Yes | Not Provided |
| Xia | 2019 | Epilepsy Behav | 2.9 | 213 | 35 | PHQ-9 (6) | 82.9 (67.3 to 91.9) | 24.6 | 84.3 (78.2 to 88.9) | 10.7 | No | Yes | Not Provided |
| Yang | 2018 | Psychol Asses | 5.1 | 612 | 44 | CES-D (30) | 84.0 (70.0 to 93.0) | 23.0 | 72.0 (69.0 to 76.0) | 7.0 | Yes | No | N/A |
| Yardeni | 2020 | Psychooncology | 3.9 | 91 | 11 | PROMIS-D (13) | 81.8 (52.3 to 94.9) | 42.6 | 82.5 (72.7 to 89.3) | 16.5 | No | Yes | Not Provided |
| Ye | 2020 | BMJ Open | 2.7 | 148 | 30 | PHQ-9 (9) | 96.7 (83.3 to 99.4) | 16.1 | 90.7 (84.1 to 94.7) | 10.6 | No | Yes | Not Provided |
| Yuan | 2019 | J Psychosom Res | 3 | 782 | 122 | PHQ-9 (10) | 86.9 (79.8 to 91.8) | 12.0 | 84.7 (81.8 to 87.2) | 5.5 | No | Yes | Not Provided |
| Zachar-Tirado | 2021 | J Head Trauma Rehabil | 2.7 | 101 | 42 | PHQ-A (5) | 90.5 (77.9 to 96.2) | 18.3 | 59.3 (46.6 to 70.9) | 24.3 | No | Yes | Not Provided |
| Zinchuk | 2020 | Epilepsy Behav | 2.9 | 175 | 76 | NDDI-E (13) | 88.2 (78.7 to 94.4) | 15.7 | 81.8 (72.8 to 88.9) | 16.1 | Yes | No | N/A |

Abbreviations: AViDI-18 = Akena Visual Depression Inventory – 18; ADAMS – Depressive Mood Subscale = Anxiety, Depression And Mood Scale; A-CSDD = Arabic version of the Cornell Scale for Depression in Dementia; BDI-I = Beck Depression Inventory-I; BDI-II = Beck Depression Inventory-II; BDI = Beck Depression Inventory; BEDS = Brief Edinburgh Depression Scale; CESD-R = Center for Epidemiologic Studies Depression Scale; CES-D = Center for Epidemiologic Studies Depression Scale; CESD-R = Center for Epidemiological Studies Depression Scale – Revised; CDST = Children Depression Screening Tool; CI = Confidence Interval; CIDT-MD = Community Informant Detection Tool for Maternal Depression; CD-RISC 10 = Connor-Davidson Resilience Scale-10; DSSS-DS = Depression and Somatic; Symptoms Scale – Depression Subscale (DS); DI-MHD = Depression Inventory for Maintenance Hemodialysis Patients; DRQ-7 = Depression Risk Questionnaire 7; DT = Distress Thermometer; EPDS-3 = Edinburgh Postnatal Depression Scale-3; EPDS = Edinburgh Postnatal Depression Scale; GDS-15 = Geriatric Depression Scale-15; GDS-4 = Geriatric Depression Scale-4; GDS-5 = Geriatric Depression Scale-5; GDS = Geriatric Depression Scale; HSCL-25 = Hopkins Symptom Checklist-25; HSCL = Hopkins Symptom Checklist; HADS-D = Hospital Anxiety and Depression Scale – Depression subscale; HADS = Hospital Anxiety and Depression Scale; HIV = Human Immunodeficiency Virus; K10 = Kessler Psychological Distress Scale-10; MGMQ = Matthey Generic Mood Questionnaire; MFQ = Mood and Feeling Questionnaire; N/A = Not Applicable; NDDI-E = Neurologic Depression Disorders Inventory in Epilepsy; NR = Not Reported; PHQ-2 = Patient Health Questionnaire-2; PHQ-9 = Patient Health Questionnaire-9; PROMIS-D = Patient Reported Outcomes Measurement Information System – Depression module; PROMIS-D-CAT = Patient Reported Outcomes Measurement Information System Depression Computer Adaptive Test; PROMIS-D-SF = Patient Reported Outcomes Measurement Information System Depression Short Form; DePreS = Post-stroke Depression Prediction Scale; PDPI-R = Postpartum Depression Predictors Inventory-Revised; PDSS = Postpartum Depression Screening Scale; RCADS-D = Revised Children’s Anxiety and Depression – Depression module; SDS = Self-Rating Depression Scale; WMH-ICS = World Mental Health-International College Student; ZLDSI = Zanmi Lasante Depression Symptom Inventory.
